# Negligible In Vitro Recovery of Macromolecules from Microdialysis Using 100 kDa Probes and Dextran in Perfusion Fluid

**DOI:** 10.1007/s11064-024-04119-7

**Published:** 2024-03-13

**Authors:** Spille Dorothee, G. Sørensen, L. R. Olsen, J. F. Bastlund, F. Sotty, D. Belling, M. H. Olsen, T. I. Mathiesen, K. Møller, F. Larsen, P. Birkeland

**Affiliations:** 1 Department of Neurosurgery, University Hospital Münster, Münster, Germany; 2 H. Lundbeck A/S, Ottiliavej 9, 2500 Copenhagen, Denmark; 3 Department of Clinical Medicine, Blegdamsvej 3, 2200 Copenhagen N, Denmark; 4 Department of Neurosurgery, Rigshospitalet, Inge Lehmannsvej 6, 2100 Copenhagen Ø, Denmark; 5 Department of Clinical Medicine, University of Copenhagen, Blegdamsvej 3, Copenhagen, Denmark; 6 Department of Clinical Neuroscience, Karolinska Institutet, Stockholm, Sweden

**Keywords:** Microdialysis, In vitro, Macromolecules, Dextran

## Abstract

Microdialysis is applied in neurointensive care to monitor cerebral glucose metabolism. If recoverable, macromolecules may also serve as biomarkers in brain disease and provide clues to their passage across the blood–brain barrier. Our study aimed to investigate the in vitro recovery of human micro- and macromolecules using microdialysis catheters and perfusion fluids approved for clinical use. In vitro microdialysis of a bulk solution containing physiological or supraphysiological concentrations of glucose, lactate, pyruvate, human IgG, serum albumin, and hemoglobin was performed using two different catheters and perfusion fluids. One had a membrane cut-off of 20 kDa and was used with a standard CNS perfusion fluid, and the other had a membrane cut-off of 100 kDa and was perfused with the same solution supplemented with dextran. The flow rate was 0.3 µl/min. We used both push and push–pull methods. Dialysate samples were collected at 2-h intervals for 6 h and analyzed for relative recovery of each substance. The mean relative recovery of glucose, pyruvate, and lactate was > 90% in all but two sets of experiments. In contrast, the relative recovery of human IgG, serum albumin, and hemoglobin from both bulk solutions was below the lower limit of quantification (LLOQ). Using a push–pull method, recovery of human IgG, serum albumin, and hemoglobin from a bulk solution with supraphysiological concentrations were above LLOQ but with low relative recovery (range 0.9%–1.6%). In summary, exchanging the microdialysis setup from a 20 kDa catheter with a standard perfusion fluid for a 100 kDa catheter with a perfusion solution containing dextran did not affect the relative recovery of glucose and its metabolites. However, it did not result in any useful recovery of the investigated macromolecules at physiological levels, either with or without a push–pull pump system.

## Introduction

Microdialysis is integrated into neurointensive care multimodal neuromonitoring alongside monitoring of intracranial pressure and brain oxygen tension [1, 2]. The underlying principle is the free diffusion of molecules across a semipermeable membrane in a double-lumen catheter. Equilibration is established after perfusion of the catheter with an isotonic fluid, and the microdialysis catheter sample reflects the composition of brain interstitial fluid [3]. Changes in brain glucose and its metabolites lactate and pyruvate may predict the development of secondary brain injury, e.g., in traumatic brain injury and delayed ischemic neurological deficits in aneurysmal subarachnoid hemorrhage [4]. Microdialysis catheters with a cut-off value of 20 kDa are in routine clinical use. These catheters easily allow the passage of small molecules such as glucose and its metabolites [5, 6]. Recently, there has been an interest in assessing larger molecules in brain interstitial fluid. For this purpose, microdialysis catheters with larger pores, i.e., 100 kDa catheters, have been developed since many pro-inflammatory molecules such as cytokines, chemokines, and immunoglobulins have a molecular weight of up to or above 100 kDa [7–9]. The cut-off value is not absolute, and even larger molecules may pass the membrane in relatively small amounts, requiring sensitive analysis for detection. Detection of complement C3 (187 kDa) and molecules between 10–232 kDa have been reported in the dialysate after clinical microdialysis with 20 and 100 kDa microdialysis probes, respectively [9, 10].

To keep perfusion fluid in the dialysate despite the larger pores, osmotic agents such as albumin or dextran have been added to the perfusion fluid. It is unknown whether using a large pore catheter with an osmotic agent affects the recovery of glucose metabolites used in routine clinical microdialysis [7]. Previously, albumin was used to increase the oncotic pressure [11], but albumin is classified as a blood product within the EU with ensuing financial and logistical problems. Therefore, dextran has largely replaced albumin [12, 13]. In this in vitro microdialysis study, we investigated the relative recovery of glucose, lactate, and pyruvate as well as large endogenous proteins (hIgG, serum albumin, and hemoglobin) at physiological and supraphysiological concentrations comparing 20 kDa probes with standard CSF fluid and 100 kDa probes with standard CSF perfusion fluid or dextran supplemented perfusion fluid.

## Methods and Material

### Microdialysis

The co-axial flow catheters with polyurethane/polyamide membranes (length 10 mm) had the membrane cut-off at molecular weights of 20 kDa (70 CMD bolt catheter, 130/10, M Dialysis AB, Stockholm, Sweden) and 100 kDa (71 high cut-off CMD bolt catheter, 130/10, M Dialysis AB,

Stockholm, Sweden). The 20 kDa microdialysis catheter was used with a standard Perfusion fluid CNS (M Dialysis AB, Stockholm, Sweden), and the 100 kDa microdialysis catheter was used with either a standard perfusion fluid CNS or perfusion fluid with an additional 3% 500-kDa molecular-weight dextran (CNS perfusion fluid with dextran, M Dialysis, Stockholm, Sweden). The experiments were run thrice using bulk solutions with physiological or supraphysiological concentrations of substances (Table 1). Each experiment was performed thrice. For the first set of experiments, a push method was used with a pump set at a flow rate of 0.3 µl/min (M Dialysis, 106 Microdialysis pump) connected at the inlet of the tubings. For the second set of experiments, a push–pull method was utilized with an additional pump at the outlet of the tubings (Microdialysis peristaltic pump, MAB 20)). The bulk solution was kept at room temperature. Microdialysate samples were collected two-hourly for 6 h and analyzed for glucose, lactate, pyruvate, and endogenous macromolecules (hIgG, serum albumin, and hemoglobin). Samples from the bulk solution were also collected at the same time points.

**Table 1:** Substances of interest, the detection range of the used assays, and concentrations in the bulk solutions – physiological concentrations determined based on CSF concentrations (What determines the CSF concentrations of albumin and plasma-derived IgG?—ScienceDirect)

Molecule	Detection range (mmol/l)	Physiological concentration (mmol/l)	Supraphysiological concentration (mmol/l)
d-glucose	0.1–25	0.2	1.5
l-lactate	0.1–12	1	4
Pyruvate	0.01–1.5	0.05	0.12
Human hemoglobin	0.000002–0.002	0.00000775	0.00155
Human serum albumin	0.000015–0.03	0.00075	0.75
Human IgG	0.00000003–0.00003	0.0000067	0.000134

### Sample Bioanalysis

Glucose, lactate, and pyruvate analyses were performed using a third-generation Microdialysis analyzer [ISCUSflex, 8003719C (mdialysis.com)]. This bed site analyzer uses enzymatic reagents and spectrophotometric measurements at 375 nm and 530 nm, respectively, and is routinely used in the neurointensive care unit. Samples were snap-frozen and stored at − 80 °C for later analysis of large molecules by mass spectrometry.

Human hemoglobin, human and bovine serum albumin, and SILu^TM^Mab (heavy-labeled human IgG used as internal standard) were purchased from Sigma-Aldrich (MO, USA). H. Lundbeck produced the human IgG standard. A standard curve containing full-length proteins in different concentrations was prepared in 1 mg/mL bovine serum albumin for each substance.

Standards and samples were processed using Waters ProteinWorks Auto-eXpress Digest kit for Hamilton STAR liquid handling system equipped with 2 Hamilton Heater Shakers (HHS), Agilent Plateloc sealer, and custom-made downholder and dark box. Mixing and heating were performed using the HHS. Plates were sealed before each incubation step using the online Agilent Plateloc sealer, and the seal was pierced using dedicated tips before the addition of the reagent.

In short, a 5 µL standard or sample was diluted with 35 µL buffer containing 0.6 µg/mL heavy-labeled internal standard (SiluMab, Sigma, MSQC6) and RapiGest provided by Waters. The samples were denatured for 10 min at 80 °C and cooled to room temperature before adding 10 µL reduction agent (DTT, provided by Waters). The samples were reduced for 20 min at 60 °C and cooled to room temperature before adding 15 µL alkylation agent (IAA, provided by Waters). The samples were placed in darkness for 30 min for alkylation. 15 µL trypsin (provided by Waters) was added to each sample, and the digest was performed at 45 °C for 2 h. Digestion is stopped by adding 4 µL inactivation solution (provided by Waters). The samples were incubated for 15 min before centrifugation at 6000×*g* at 5 °C for 20 min. Samples from bulk solution were diluted 25 times before analysis to match the range of the standard curves.

All analytical work was done using the Acquity UPLC system (Waters, MA, USA) coupled with Xevo TQ-S triple quadrupole (Waters, MA). Separation was performed by injecting 20 µL supernatant onto an Acquity CSH C18 Column, 130 Å, 1.7 μm, 2.1 × 100 mm (Waters, MA) using an 8-min gradient elution at a column temperature of 55 °C. The mobile phases consisted of 0.1% formic acid in MilliQ water (A) and 0.1% formic acid in acetonitrile (B) at a flow rate of 0.4 mL/min. The gradient started after 2 min at 2% B and increased to 30% B at 8 min. Then, the column was washed with 90% B for 1 min before returning to 2% B for 2 min for equilibration. The mass spectrometer was operated in positive ESI mode and set to a scheduled MRM method with the settings listed in Table 2. The transition highlighted in bold was used for quantification. The MS settings are listed in Table 3.

**Table 2:** MS transitions for hIgG quantification

Protein	Name	Q1 (charge state)	Q3	Cone voltage (V)	Collision energy
hIgG	ALPA	419.76 (2^+^)	654.38	20	11
			486.29	20	20
	DTLM	418.22 (2^+^)	506.28	20	17
			619.36	20	16
	GPSV	593.83 (2^+^)	699.40	40	21
			846.47	40	21
	VVSV	603.34 (3^+^)	712.39	20	21
Heavy-labeled hIgG	^C13N15^ALPA	423.76 (2^+^)	662.40	20	11
			494.31	20	20
	^C13N15^DTLM	423.23 (2^+^)	516.28	20	17
			629.37	20	16
	^C13N15^GPSV	597.83 (2^+^)	707.42	40	21
			854.49	40	21
	^C13N15^VVSV	606.01 (3^+^)	716.40	20	21
HSA	LVNEVTEFAK	575.3 (2^+^)	937.5	25	20
HHG	VNVDEVGGEALGR	657.8 (2^+^)	659.3	25	25

**Table 3:** MS settings

Xevo TQ-S:	
Capillary (V)	3
Source offset (V)	50
Source temperature (°C)	150
Desolvation temperature (°C)	400
Cone gas flow (L/Hr)	150
Desolvation gas flow (L/Hr)	900
Collision gas flow (L/Hr)	0.15
Nebuliser gas flow (mL/min)	7.00

Quantification was performed using TargetLynx® software (Waters, MA, USA).

For hIgG, the response of the peptide ALPAPIEK was used as quantifier peptides, and DTLMISR, GPSVFPLAPSSK, and VVSVLTVHQDWLNGK were used as qualifier peptides. HSA and HHG were quantified using the surrogate peptides in Table 2 with the same internal standard used for hIgG quantification. At least 5 standards were included in the standard curve, and the concentration of the molecules was calculated from this curve using linear regression and 1/x weighting. The calculated concentrations in the QC samples were all within 20% of the nominal value.

### Data Analysis

Data were analyzed using GraphPad Prism 9. Data are presented as mean (± SEM) for dialysate and bulk concentrations of the examined molecules at 2 h, 4 h, and 6 h. In vitro recovery (relative recovery (%) = (dialysate concentration/test solution concentration) × 100) was determined by measurement of the substance concentration in the dialysate fluid as well as in the bulk solution. The relative recovery of each molecule was calculated as a mean across all time points.

## Results

### Glucose, Lactate, and Pyruvate

**Fig. 1:**
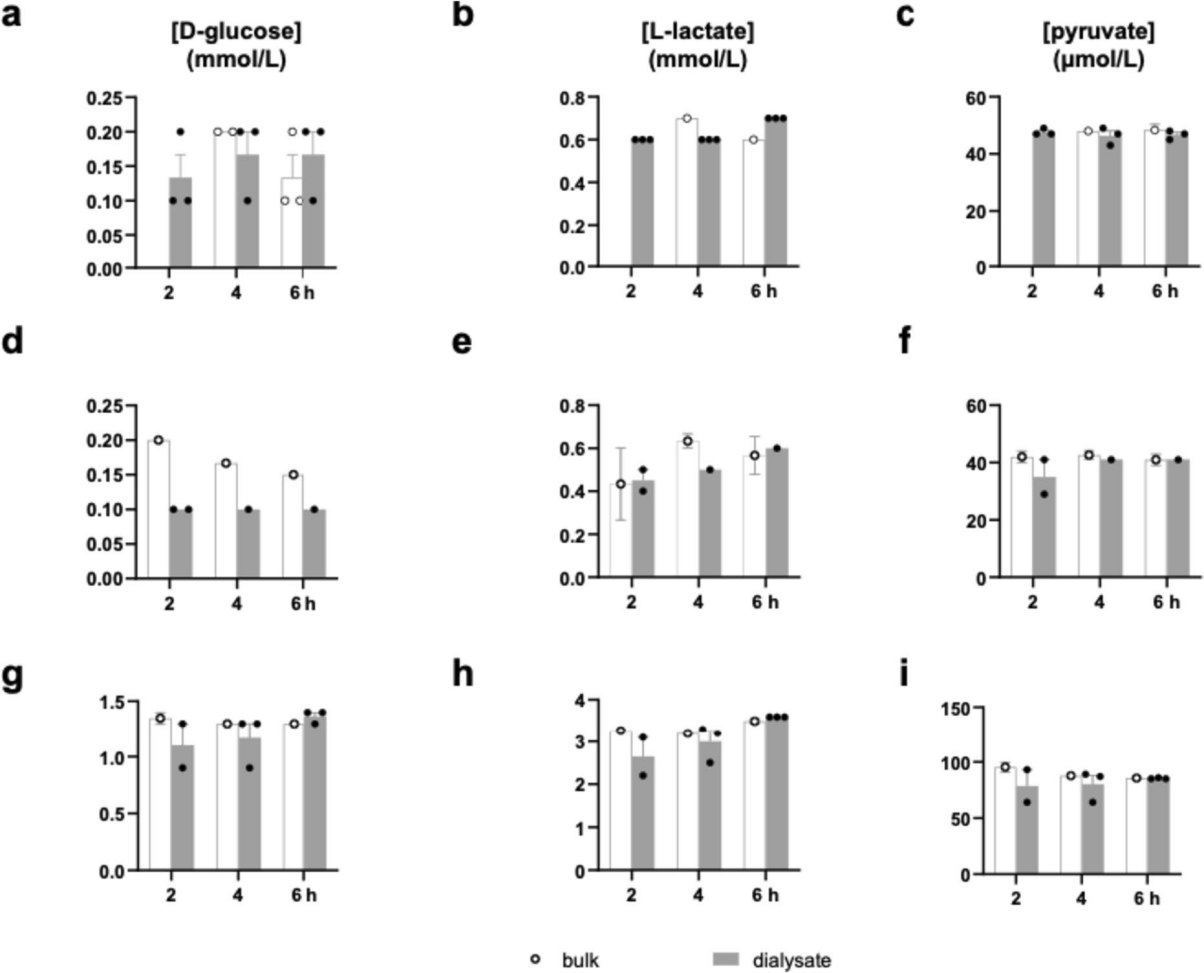
Levels of d-glucose (**a, d, g**), l-lactate (**b, e, h**), and pyruvate (**c, f, i**) measured in bulk solution at 4 and 6 h and dialysate at 2, 4, and 6 h following initiation of the perfusion. **a–c** Dialysates were obtained from 20 kDa catheters perfused with standard fluid and using a bulk solution containing physiological concentrations of the metabolites of interest; **d–i** Dialysates were obtained from 100 kDa catheters perfused with fluid with an additional 3% 500-kDa molecular-weight dextran and using a bulk solution containing either physiological concentrations (**d–f**) or high concentrations (**g–i**) of the metabolites of interest. Data are presented as mean ± SEM

The mean dialysate concentrations of glucose measured by the 20 kDa catheter were 0.13 mM (± 0.03) after 2 h and 0.17 (± 0.03) mM after 4 h and 6 h, respectively (Fig. 1a). Mean dialysate concentrations of glucose as measured by the 100 kDa-catheters with a perfusion fluid with an additional 3% 500-kDa molecular-weight dextran applying a physiological or supraphysiological concentrations of substances in the bulk solution were similar [0.10 (single data point) mM] as well as 1.10 (± 0.2) mM, 1.17(± 0.13) mM and 1.37(± 0.03) mM after 2 h, 4 h, and 6 h (Fig. 1d, g). The 100 kDa-catheters with a standard perfusion fluid did not yield any dialysate and were thus abandoned.

The mean dialysate concentrations of lactate measured by the 20 kDa-catheter were 0.60(± 0.00) mM after 2 h and 4 h, respectively, and 0.70 (± 0.00) mM after 6 h (Fig. 1b). Mean dialysate concentrations of lactate as measured by the 100 kDa-catheters applying a physiological concentration of molecules in the bulk solution were 0.45 (± 0.05) mM, 0.50 (± 0.00) mM, and 0.60 (± 0.00) mM. Using a supraphysiological concentration in the bulk solution, mean dialysate concentrations of lactate as measured by the 100 kDa-catheter were 2.65 (± 0.45) mM, 3.00 (± 0.25) mM, and 3.60 (± 0.00) mM after 2 h, 4 h, and 6 h, respectively (Fig. 1e, h).

Mean pyruvate dialysate concentrations measured by the 20 kDa-catheter were 47.67 (± 0.67) µM, 46.33 (± 1.76) µM and 46.67 (± 0.88) µM after 2 h, 4 h, and 6 h, respectively (Fig. 1c). Using the 100 kDa-catheter with physiological concentration of substances in the bulk solution, the mean dialysate concentrations amounted to 35.00 (± 6.00) µM, 41.00 (± 0.00) µM and 41.00 (± 0.00) µM. Utilizing the 100 kDa-catheter with supraphysiological concentration of the bulk solution, the mean dialysate concentrations were 78.50 (± 14.50) µM, 80.00 (± 8.02) µM and 85.33 (± 0.33) µM following 2 h, 4 h, and 6 h, respectively (Fig. 1f, i).

The mean relative recovery of glucose, pyruvate, and lactate, respectively, was > 90% in all but two sets of experiments: The relative recovery of glucose in a physiological concentration using a 100 kDa catheter was 58%, and the relative recovery of lactate using af 20 kDa catheter was 81%.

### Human IgG, Human Serum Albumin, and Human Hemoglobin

**Fig. 2:**
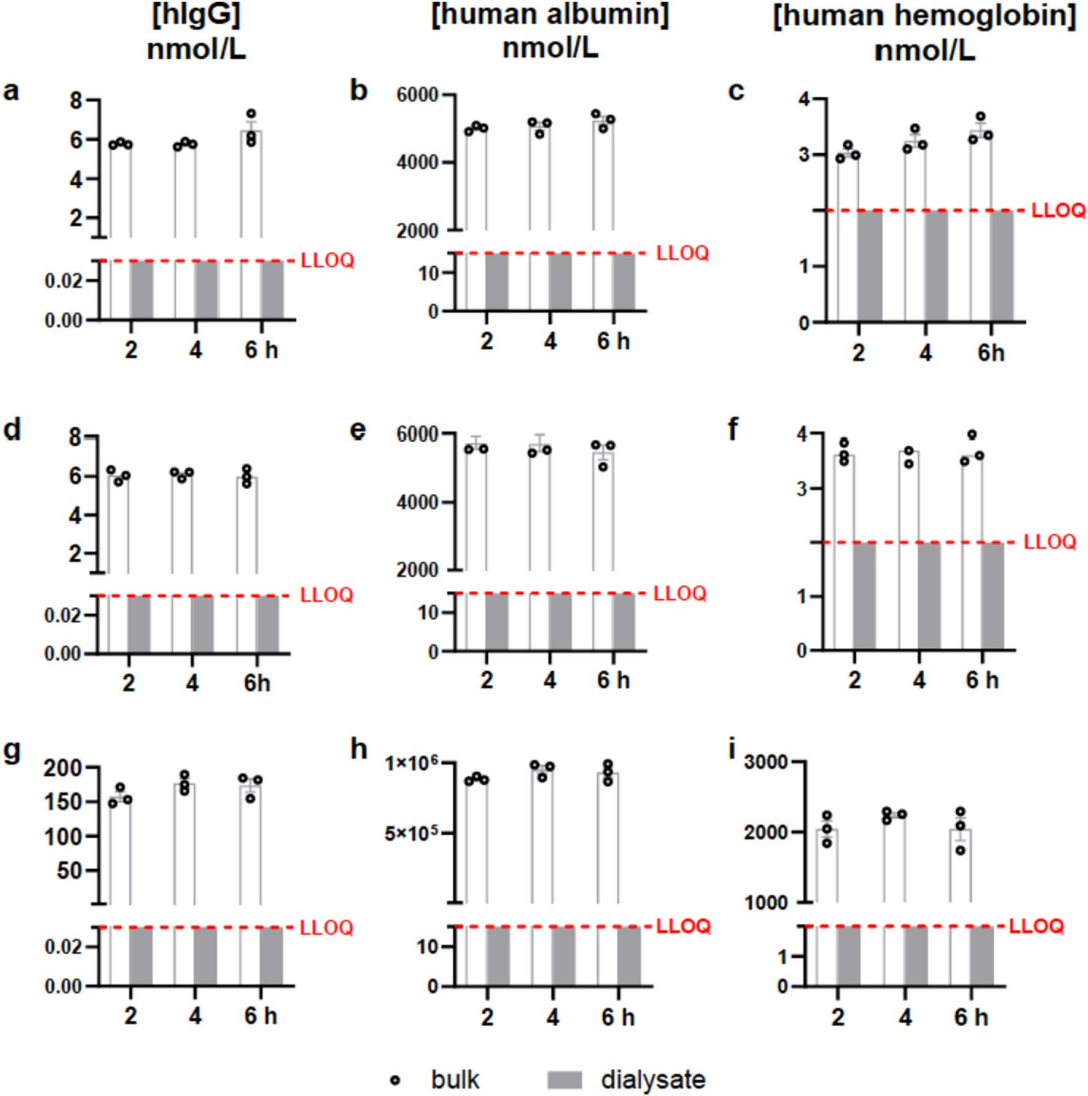
Levels of human IgG (**a, d, g**), human albumin (**b, e, h**), and human hemoglobin (**c, f, i**) measured in bulk solution at 2, 4, and 6 h following initiation of the perfusion. The analysis level was below the lower limit of quantification (LLOQ) in all conditions. **a–c** Dialysates were obtained from 20 kDa catheters perfused with standard fluid and using a bulk solution containing physiological concentrations of the proteins of interest; **d–i** Dialysates were obtained from 100 kDa catheters perfused with fluid with an additional 3% 500-kDa molecular-weight dextran and using a bulk solution containing either physiological concentrations (**d–f**) or high concentrations (**g–i**) of the proteins of interest. Data are presented as mean ± SEM

**Fig. 3:**
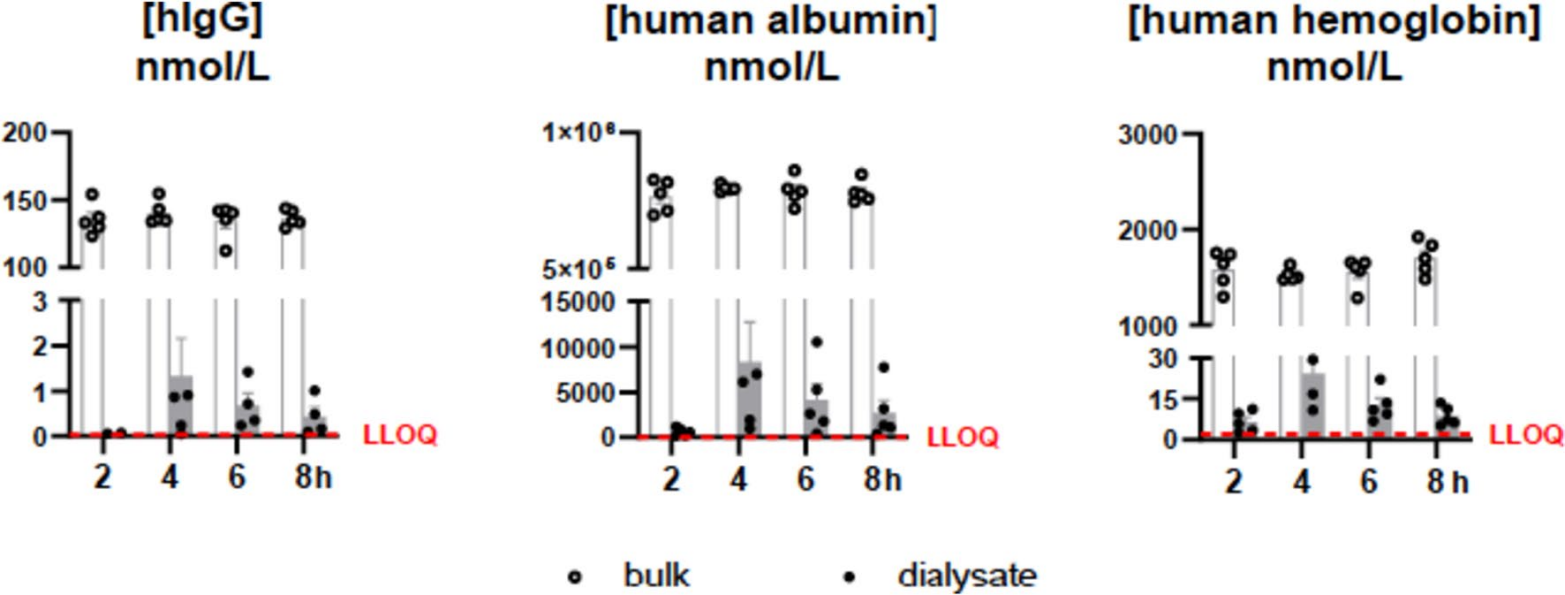
Levels of human IgG, human albumin, and human hemoglobin measured in bulk solution and dialysates at 2, 4, 6, and 8 h following initiation of the perfusion using the push–pull method. Dialysates were obtained from 100 kDa catheters perfused with fluid with an additional 3% 500-kDa molecular-weight dextran using a bulk solution containing high concentrations of the proteins of interest. Data are presented as mean ± SEM

Data on transmembrane fluid balance for the 20 kDa- and 100 kDa-catheters are shown in Figs. 2 and 3. Using either the 20 kDa-catheter without dextran or the 100 kDa-catheter with dextran, the mean dialysate concentrations for IgG, albumin, and hemoglobin were below the lower limit of quantification (LLOQ) as the lowest concentration in the standard curve (Fig. 2a–f). Likewise, when applying a bulk solution with supraphysiological concentrations, no values above the LLOQ were measured in the dialysate solution (Fig. 2g–i). Thus, this experimental setup could detect no relative recovery for the macromolecules studied. Based on the different LLOQs and the concentration in the bulk solution, the relative recoveries are below 0.1% for hemoglobin, 0.025% for hIgG, and 0.000002% for albumin.

Applying a push–pull microdialysis technique with large pore membranes (100 kDa) with dextran mean dialysate concentrations for IgG, Albumin, and hemoglobin amounted to concentrations at 4 h of 1.3 nM, 550 nM, and 24.3 nM, respectively (Fig. 3)*.* Each protein’s in vitro relative recoveries yielded 0.9%, 1%, and 1.6% at 4 h. The recovery was time-dependent, increasing from 2 to 4 h and declining at 6 and 8 h.

## Discussion

Our main finding is that push–pull is necessary to recover large molecules in the dialysate; however, it still has a very low recovery. In addition, there is an issue with stability over time. Notably, recovery was only possible with supraphysiological concentrations of molecules in the bulk solution, and detecting large molecules at physiological concentration would be very difficult based on the low recovery and LLOQ of the assay for detection. As a secondary finding, our in vitro study showed that using 100 kDa probes and CNS perfusion fluid with dextran did not affect the measurement of glucose and its metabolites compared to 20 kDa probes with standard perfusion fluid in routine clinical use.

*Relative recovery of small molecules—glucose, pyruvate, and lactate* Some centers routinely use microdialysis (20 kDa catheters with standard CNS perfusion fluid) in neurointensive care patients with traumatic brain injury or aneurysmal subarachnoid hemorrhage to monitor glucose metabolism. These small molecules offer a snapshot of energy metabolism but fail to discriminate underlying mechanisms such as inflammation. Larger proinflammatory molecules, such as cytokines and chemokines, have been targeted as possible pathogenic mediators [7, 9, 14]. To monitor these molecules, catheters with larger pore sizes are necessary [7, 14], while measurement of small molecules must not be affected. The recovery of glucose, pyruvate, and lactate—with molecular weights of approximately 180 Da, 87 Da, and 90 Da, respectively [1, 2]—has been widely studied and well-established in clinical practice [4–6]. Hutchinson et al. [15] demonstrated equal relative recovery for small molecules using the 20 kDa and 100 kDa catheters under in vitro and in vivo conditions but did not validate the use of dextran. In our in vitro study, the measurements of solutes showed comparable values for relative recovery of glucose, pyruvate, and lactate using either 20 kDa catheters and standard perfusion fluid or 100 kDa catheters and perfusion fluid with dextran. In contrast, Hillmann et al. [16] simultaneously implanted 20 kDa and a 100 kDa catheter in 15 comatose patients and found significant differences in lactate recovery despite the nearly equal recovery of glucose and pyruvate. The differences may reflect differences in dialysate since the standard solution was used for the 20 kDa setup. In contrast, RingerDextran solution with a mixture of chloride and acetate anions was used for 100 kDa microdialysis. It is possible that acetate and lactate interactions affected recovery and that the observed difference was due to different solutes rather than the different membranes. Our in vitro data fill a knowledge gap and validate the interchangeability of 20 kDa catheters with standard CNS perfusion fluid and 100 kDa catheters with dextran-added CNS perfusion fluid in microdialysis for measuring glucose and metabolites.

### Assessment of Dextran as Perfusion Fluid

In our study, microdialysis using 100 kDa catheters and standard perfusion fluid did not return any perfusate, probably due to fluid leaking through the larger pores. The oncotic pressure in the perfusion fluid can be increased by using colloids to minimize this phenomenon [11], e.g., dextran [13, 17, 18]. Overall, using 500 kDa Dextran perfusate reduced leakage of perfusion fluid and improved recovery. Giorgi-Coll and colleagues [12] reported improved fluid and cytokine recovery using 3% dextran 500 kDa perfusion fluid compared to conventional perfusion fluid. In our study, the use of perfusion fluid with dextran was necessary for generating samples of perfusate.

*Relative recovery of macromolecules—IgG, albumin, and hemoglobin* To provide recovery of macromolecules, the use of catheters with membranes with increased molecular weight cut-off has been examined in vitro and in vivo [7, 8]. Bergman and colleagues [9] used a 100 kDa microdialysis catheter with an aqueous solution supplemented with dextran (30 g dextran-60 1000 ml ^−1^) as perfusion fluid in 10 patients with progressive multiple sclerosis to evaluate the mechanism of action of rituximab, a monoclonal antibody. One hundred eighty proteins with molecular weights ranging from 10 to 232 kDa were examined using a multiplex immunoassay. An increase in molecular weight was associated with decreased relative recovery, but partial recovery was described even for molecules above 100 kDa. This surprising finding disagrees with our results. It is possible that the semi-quantitative method with protein amplification in real-time PCR was more sensitive to detecting minimal amounts of large molecules. If so, repeatability will be affected since recovery must be very low; we could not measure any recovery of macromolecules with either the 20 kDa or the 100 kDa catheter. Immunoglobulin G, serum albumin, and hemoglobin have molecular weights of approximately 150 kDa, 66 kDa, and 64 kDa. Thus, albumin and hemoglobin would not necessarily be too large to pass the membrane. However, other chemical properties besides molecular weight influence the recovery of the molecules, e.g., protein aggregation, shape, surface charge, hydrophobicity, probe perfusion rate, and duration [19, 20]. Hemoglobin may show low relative recovery because its subunits display high membrane absorption, e.g., for microdialysis, due to their ability to bind oxygen [21, 22]. Similarly, Chang et al. [23] could affirm the non-specific absorption of IgG to the microdialysis probes and pointed out molecular weight, net charge, and ability to interact with Fc-receptors and ultrafiltration using large pore catheters as further limitations to antibody recovery. The low relative recovery of albumin is not primarily due to its molecular weight but to its property of binding and transporting endogenous ligands [24]. Hydrophobic interactions are most likely to impair the crossing of albumin [25, 26]. Importantly, the recovery was independent of macromolecular concentrations in the bulk solution, which agrees with previous findings [27, 28]. In summary, our observations disagree with earlier reports of relevant recovery of large molecules with 100 or 20 kDa microdialysis catheters. Future studies must clarify to which extent quantitative data on the recovery of macromolecules are reproducible.

A literature search indicates that larger 1 MDa pore probes are necessary for reproducible recovery of large molecules [23, 29–33]. Larger pore-size catheters have yet to be approved for clinical use.

### Limitations

Our study has some limitations. The analyses were performed in vitro, which allows extensive experimental control but may introduce unknown sources of error that limit application in vivo. Furthermore, the macromolecules we investigated have a high molecular weight (approximately 64–150 kDa), which is a theoretical obstacle to significant passage across the microdialysis membranes and, together with possible molecular interactions with membranes and dextran, may have resulted in concentrations too low to quantify in our dialysate reliably.

## Conclusions

In conclusion, catheters with a membrane cut-off of 20 kDa and 100 kDa yielded similar results of relative recovery of small molecules. A perfusion solution with high osmolality should be applied when using the 100 kDa catheters, and dextran was suitable for this purpose. We could not demonstrate useful recovery of large molecules at physiological concentrations. Detailed protocols for microdialysis of large molecules should be validated in vitro to secure clinical reproducibility.

## Data Availability

The data supporting this study’s findings are available from the corresponding author upon reasonable request.

## Abbreviations

LLOQ: Lower limit of quantification
CNS: Central nervous system
hIg: Human immunoglobulin
CSF: Cerebrospinal fluid
SEM: Standard error of the mean
PCR: Polymerase chain reaction
